# Whole-Mount Kidney Clearing and Visualization Reveal Renal Sympathetic Hyperinnervation in Heart Failure Mice

**DOI:** 10.3389/fphys.2021.696286

**Published:** 2021-07-08

**Authors:** Chao Wu, Fang Yan, Min Li, Yimin Tu, Ziyu Guo, Yufei Chen, Yaxin Wu, Qing Li, Changan Yu, Yi Fu, Meihui Wu, Wei Kong, Yanxiang Gao, Xiaowei Li, Jingang Zheng

**Affiliations:** ^1^Department of Cardiology, Peking University China-Japan Friendship School of Clinical Medicine, Beijing, China; ^2^School of Biomedical Engineering, Shanghai Jiao Tong University, Shanghai, China; ^3^Graduate School, Chinese Academy of Medical Sciences and Peking Union Medical College, Beijing, China; ^4^Department of Cardiology, China-Japan Friendship Hospital, Beijing, China; ^5^Department of Physiology and Pathophysiology, School of Basic Medical Sciences, Peking University, Beijing, China; ^6^Key Laboratory of Molecular Cardiovascular Science, Ministry of Education, Beijing, China; ^7^Department of Nursing, Army Medical Center of PLA, Chongqing, China

**Keywords:** renal dysfunction, tissue clearing, CUBIC, 3D imaging, heart failure, sympathetic nerve

## Abstract

Developing a three-dimensional (3D) visualization of the kidney at the whole-mount scale is challenging. In the present study, we optimized mouse whole-mount kidney clearing, which improved the transparency ratio to over 90% based on organ-specific perfusion (OSP)-clear, unobstructed brain imaging cocktails and computational analysis (CUBIC). The optimized OSP-CUBIC-compatible 3D immunostaining and imaging simultaneously visualized the high-resolution 3D structure of the whole-mount renal microvascular, glomerulus, and accompanying wrapped traveling sympathetic nerves in mice. A mouse model of pressure overload-induced heart failure (HF) was then established by minimally invasive transverse aortic constriction (MTAC). Further 3D quantification revealed renal sympathetic hyperinnervation (6.80 ± 1.04% *vs*. 3.73 ± 0.60%, *P* < 0.05) in mice with HF. In conclusion, this newly developed whole-organ tissue clearing and imaging system provides comprehensive information at the whole-mount scale and has great potential for kidney research. Our data suggest that renal sympathetic hyperinnervation is involved in HF associated with renal dysfunction.

## Introduction

Kidney dysfunction is among the most common coexisting conditions in patients with acute or chronic heart disease (Conrad et al., 2018). The term cardiorenal syndrome (CRS) encompasses a spectrum of disorders that involve both the heart and the kidney, in which acute or chronic dysfunction in one organ can induce abnormalities in another organ (Rangaswami et al., 2019). In clinical practice, over 50% of patients with acute decompensated heart failure (HF) have some degree of renal insufficiency (Heywood et al., 2007).

The conventional explanation for the development of CRS in the setting of cardiac disorders focuses on the inability of the failing heart to generate forward flow, thus resulting in prerenal hypoperfusion. Inadequate renal afferent flow activates the sympathetic nervous system (SNS), the renin–angiotensin–aldosterone system (RAAS) axis, and arginine vasopressin secretion, eventually leading to fluid retention, higher preload, and worsening pump failure (Schrier and Abraham, 1999; Savira et al., 2020). However, the presence of a low-flow state only partially explains the pathophysiology of CRS. SNS hyperactivation is a major compensatory mechanism to maintain inotropic support and cardiac output. In the progression of chronic kidney disease (CKD) deterioration, SNS is over-activated in response to renal ischemia; decreased nitric oxide and increased angiotensin II levels result in SNS over-activation and subsequently lead to hypertension and left ventricular (LV) hypertrophy/dilatation (Kumar et al., 2014). In addition, persistent activation of myocardial β1-adrenoceptors leads to impaired receptor-signal transduction, which may result in cardiac dysfunction (Hatamizadeh et al., 2013). More studies are needed to illustrate the role of SNS with CRS condition.

Histological techniques have been the standard procedure for investigating various tissues for decades. However, a complete understanding of biological mechanisms in both health and disease requires an unbiased exploration of the whole organism and not simply selected thin tissue sections. To avoid these limitations, tissue clearing methods that allow the three-dimensional (3D) imaging of intact tissues and even some entire organisms have been developed in recent years. Three major tissue-clearing approaches are currently available: hydrophobic, hydrophilic, and hydrogel-based (Susaki and Ueda, 2016; Tainaka et al., 2016). These tissue-clearing methods are generally able to remove lipids (delipidation), pigments (decolorization), and calcium phosphate (decalcification) highly efficiently to reach almost a complete level of transparency for some intact organs and even entire adult rodent bodies (Ueda et al., 2020). Hydrophobic tissue-clearing methods [e.g., benzyl alcohol and benzyl benzoate (BABB), 3D imaging of solvent cleared organs (3DISCO), ultimate DISCO (uDISCO), immunolabeling-enabled DISCO (iDISCO)] (Dodt et al., 2007; Erturk et al., 2011, 2012; Pan et al., 2016) render high-transparency organs within a few days. However, the cleared organ will shrink, which is one disadvantage for high-resolution imaging. Hydrogel-based methods [e.g., clear lipid-exchanged acrylamide-hybridized rigid imaging/immunostaining/*in situ*-hybridization-compatible tissue hydrogel (CLARITY)] (Chung et al., 2013) provide high-transparency organs within 1 week, but this method is difficult to apply to many samples without dedicated electrophoresis equipment. Clear, unobstructed brain imaging cocktails and computational analysis (CUBIC) (Susaki et al., 2014; Tainaka et al., 2014) reagents that are based on hydrophilic reagents have been developed to prioritize safety and decrease environmental burden. These protocols require only sample immersion in clearing media, which mitigates operation difficulty. Notably, the kidney is a heme-rich organ, which hampers complete tissue clearing and subsequent whole-organ imaging. The amino alcohols in CUBIC reagents have the potential to make samples render higher decolorization transparency (Susaki et al., 2014; Tainaka et al., 2014).

To better understand renal abnormalities and regulation in the whole-mount kidney, we developed a method of whole-organ tissue clearing that is compatible with 3D immunostaining, 3D imaging, and analysis to show clear macro- and microstructures of the whole-mount mouse kidney. Sympathetic hyperinnervation of the kidney in pressure overload-induced HF was observed by applying an optimized whole-organ clearing and imaging protocol.

## Materials and Methods

### Experimental Design

Randomization: Each experimental mouse had a unique number that was generated by the “RAND” function in Microsoft Excel, and the mice were divided into the study groups in numerical order of the unique number.

Blinding: All of the experimental data were verified by an independent investigator who was blinded to the study group.

Number of replicates: All of the experiments included biological replicates. The number of samples in each experiment is described in each figure legend. For all of the experiments, control data were acquired concurrently with data in which statistical comparisons were performed.

### Experimental Animals

Male adult (8–12 week old) C57BL/6N mice were purchased from Beijing Vital River Laboratory. The mice were maintained on a 12/12 h light/dark cycle and fed with a normal laboratory rodent diet. All of the mice were maintained in an animal facility that was approved by the Association for the Assessment and Accreditation of Laboratory Animal Care at China-Japan Friendship Hospital. All of the animal experiments were approved by the Institutional Animal Care and Use Committee of China-Japan Friendship Hospital and complied with all relevant ethical regulations.

### Optimized Whole-Organ Clearing

The organ-specific perfusion-CUBIC (OSP-CUBIC) clearing protocol was optimized based on the conventional CUBIC protocol (Susaki et al., 2015; Matsumoto et al., 2019). Briefly, before anesthesia, the mice received an intraperitoneal injection of 10 ml/kg heparin (1000 U/ml). After the mice were anesthetized, the thoracic and abdominal cavity was exposed, and the inferior renal vascular, brachiocephalic trunk, left common carotid artery, and left subclavian artery were ligated. The arteries were then transcardially perfused with 20 ml of cold heparin-phosphate-buffered saline (PBS; 10 U/ml) with a peristaltic pump perfusion velocity below 10 ml/min. Blood was removed from the tissues as much as possible, followed by 20 ml of the vascular dye FluoSphere (catalog no. F8801, ThermoFisher Scientific, Waltham, MA, United States) diluted in cold heparin-PBS, 150 ml of cold 4% (wt/vol) paraformaldehyde, 100 ml of CUBIC-P [5 wt% 1-methylimidazole (catalog no. M0508, Tokyo Chemical Industry, Tokyo, Japan), 10 wt% *N*-butyldiethanolamine (catalog no. B0725, Tokyo Chemical Industry, Tokyo, Japan), and 5 wt% Triton X-100 (catalog no. X100, Sigma-Aldrich, St. Louis, MO, United States)]. After perfusion with various reagents, the kidney was dissected from the mouse and immersed in 10 ml of 1/2 CUBIC-L (10% wt/wt *N*-butyldiethanolamine and 10% wt/wt Triton X-100 in ddH_2_O, 1:1 dilution with ddH_2_O) in a 50 ml tube overnight at 37°C with gentle shaking. The next day, 10–15 ml of CUBIC-L was replaced in the 50 ml tube and delipidated/decolorated for another 5 days at 37°C with gentle shaking. After tissue clearing, the sample was washed in PBS for 2 h (three times) at room temperature with gentle shaking. The washing tube was replaced after each washing step to completely remove Triton X-100. The sample was then used for 3D immunostaining, refractive index (RI) matching, or temporary storage. For temporary storage, the kidney was immersed in 10 ml of 40% (wt/vol) sucrose in PBS with shaking at room temperature overnight. When the samples sank to the bottom, they were placed in O.C.T. compound and immediately stored at −80°C.

### Three-Dimensional Immunostaining

For 3D immunostaining of whole-mount kidney sympathetic nerves, OSP-CUBIC-cleared kidneys were sufficiently washed with PBS solution to remove clearing reagents. The kidney was first immersed in 5 ml staining buffer (10 mM HEPES, 10% triton X-100, 200 mM NaCl, and 0.5% w/v bovine serum albumin in ddH_2_O) for 2 h at 37°C with gentle shaking. The kidney was then removed to 500 μl staining buffer with 1:200 anti-Tyrosine hydroxylase (TH) antibody (catalog no. ab112, Abcam, Cambridge, MA, United States) for 7 days at 37°C with shaking. After washing with 1% PBST (1% Triton X−100), the kidney was incubated with 500 μl staining buffer with secondary antibody conjugated to Alexa Fluor 647 (catalog no. ab32733, Invitrogen, Carlsbad, CA, United States) for 7 days at 37°C. After washing with 1% PBST for several hours, the samples were immersed in 1% paraformaldehyde overnight at room temperature, followed by another three cycles of PBS washing. The kidney was then placed in 5 ml of 1/2 CUBIC-R + (N) [45% wt/wt antipyrine (catalog no. D1876, Tokyo Chemical Industry, Tokyo, Japan), 30% wt/wt nicotinamide (catalog no. N0098, Tokyo Chemical Industry, Tokyo, Japan), and 25% wt/wt ddH_2_O, and 0.5% vol/vol *N*-butyldiethanolamine in a 1:1 dilution with ddH_2_O] in a 10 ml tube overnight at 37°C with gentle shaking. The next day, 5 ml of CUBIC-R + (N) was replaced in the 10 ml tube and incubated for another 4 days.

### Light-Sheet Fluorescence Microscopy Imaging

The cleared whole-mount kidney was imaged using a UltraMicroscope II (LaVision BioTec GmbH, Bielefeld, Germany) that was equipped with a white-light laser module (NKT SuperK Extreme EXW-12), six fixed light-sheet generating lenses, an sCMOS camera (2560 × 2160, 6.5 μm pixel size, Andor Neo), and a 2 × objective lens (Olympus MVPLAPO) covered with a 6 mm working distance dipping cap. Sixteen-bit optical sectional images of two channels (excitation at 561 and 640 nm, respectively) were acquired with a total magnification of 1.26 (N.A. = 0.14) with a z-step of 5 μm. During image acquisition, the optically cleared sample was immersed in an RI-matched oil mixture of silicon oil HIVAC-F4 (RI = 1.555, catalog no. HIVAC-F4, Shin-Etsu Chemical, Tokyo, Japan) and mineral oil (RI = 1.467, catalog no. M8410, Sigma-Aldrich, St. Louis, MO, United States).

### Confocal Microscopy Imaging

The confocal images were acquired using a Leica TCS SP8 microscope (Leica Microsystems GmbH, Wetzlar, Germany) that was equipped with a 25 × objective (N.A. = 0.95), with a voxel size of 0.8 μm × 0.8 μm × 1.98 μm. Two color fluorescence images with excitation wavelengths of 561 and 640 nm were taken for imaging blood vessels and sympathetic nerves, respectively. The cleared kidney was immersed in the RI-matched solution during imaging as described above.

### Image Processing of Light-Sheet Fluorescence Microscopy Images

To quantify the 3D immunofluorescence information, the fluorescent image stacks that were acquired using light-sheet fluorescence microscopy (LSFM) were processed as follows. Background subtraction was performed, and then the images were segmented using the surface module in Imaris 9.6 (Oxford Instruments, Abingdon, United Kingdom) to calculate the TH signal-positive volume. To calculate kidney volume, the fluorescent images were segmented with the Li algorithm (Li and Lee, 1993; Li and Tam, 1998) in ImageJ 1.52n software (National Institutes of Health, Bethesda, MD, United States). The segmented images were further processed using a median filter (Boyle and Thomas, 1988; Davies, 1991) and fill hole operations in ImageJ to obtain binary images of the whole kidney. Finally, the total volume of the kidney was obtained by summing the volumes of all sections in the image stacks using MATLAB (2019a, MathWorks, Natick, MA, United States).

### Minimally Invasive Transverse Aortic Constriction

Minimally invasive transverse aortic constriction (MTAC) was surgically induced as previously described (Zaw et al., 2017). After the mice were anesthetized, hair was removed on the chest and anterior neck with depilatory cream. Ophthalmic gel was applied to the animal’s eyes to prevent the cornea from drying out. The surgical site was disinfected with 75% ethyl alcohol. The mouse was placed on a heating pad with the temperature adjusted to 37°C. The skin was opened at the midline of the neck and chest with a scalpel. Connective tissues were gently separated with blunt scissors, and the sternum was cut to the second rib (approximately 5 mm). The thymus lobes were carefully separated from one another and the lower chest wall by separating the connective tissue with a curved needle. The transverse aortic arch and two carotid arteries were then clearly visible. A 6-0 monofilament suture was placed under the aortic arch, and a curved 27-gauge needle (0.417 mm spacer) was placed in the loop. The suture was fixed in place with a double-knot. The spacer was gently removed, and the suture ends were cut. The chest wall and skin were closed with a 6-0 suture and 5-0 suture, respectively. After the animal recovered from anesthesia, it was returned to the breeding room.

### Echocardiography

Transthoracic echocardiography was performed using a VisualSonics Vevo 1100 system that was equipped with an MS400 transducer (Fujifilm VisualSonics, Toronto, ON, Canada). After 1 week, the previously experimental mouse was anesthetized with isoflurane. After depilating hair, the mouse was secured in the supine position on a platform. Ultrasound gel was then applied to the mouse’s chest. Using an MS400 probe and a high-frequency ultrasound system, color Doppler and pulsed-wave Doppler scanning were performed on the left and right carotid arteries and aortic arch. Blood flow of the left common carotid artery, right carotid artery, and constricted site was evaluated.

Systolic function was measured 0, 1, 4, 6, and 8 weeks after MTAC surgery or sham surgery. Indices of systolic function were obtained from short-axis M-mode scans at the midventricular level, indicated by the presence of papillary muscles. During echocardiogram acquisition, heart rate was maintained at 450–550 beats per min. All parameters were measured at least three times, and means are presented.

### Histological Examination

The kidneys were collected and fixed in 4% paraformaldehyde overnight and processed for routine paraffin histology [5 μm sections stained with hematoxylin and eosin (H&E) and Masson’s trichrome]. The renal fibrotic area was quantified in three microscopic fields per kidney using ImageJ 1.52n software (National Institutes of Health, Bethesda, MD, United States).

### Measurement of BNP, CRE, and BUN

The levels of brain natriuretic peptide (BNP), creatinine (CRE), and blood urea nitrogen (BUN) in plasma samples were measured using the BNP enzyme-linked immunosorbent assay kit (catalog no. DG30180M, Dogesce, Beijing, China), CRE assay kit (catalog no. C011-2-1, Nanjing Jiancheng Bioengineering Institute, Nanjing, China), and BUN assay kit (catalog no. C013-2-1, Nanjing Jiancheng Bioengineering Institute, Nanjing, China) according to the manufacturer’s instructions.

### Measurement of 24-h Urine Albumin

The level of urine albumin was measured using the mouse albumin enzyme-linked immunosorbent assay kit (catalog no. E99-134, Bethyl Laboratories Inc., Montgomery, TX, United States), according to the manufacturer’s instructions. Twenty-four-hour mouse urine was collected with mouse metabolic cage while restricting food.

### Statistical Analysis

All of the results are expressed as mean ± standard error of the mean. For statistical comparisons, we applied two-tailed unpaired Student’s *t*-test or two-way analysis of variance for comparisons between two groups. In all cases, two-tailed probability less than 0.05 (*P* < 0.05) was considered statistically significant. The details of each statistical analysis for each experiment are presented in the corresponding figure legends. The statistical analyses were conducted using Prism 8.0 software (GraphPad, San Diego, CA, United States) or SPSS Statistics 26.0 software (IBM, Armonk, NY, United States).

## Results

### Optimized OSP-CUBIC Clearing Renders Whole-Mount Kidney With High Transparency

**FIGURE 1:**
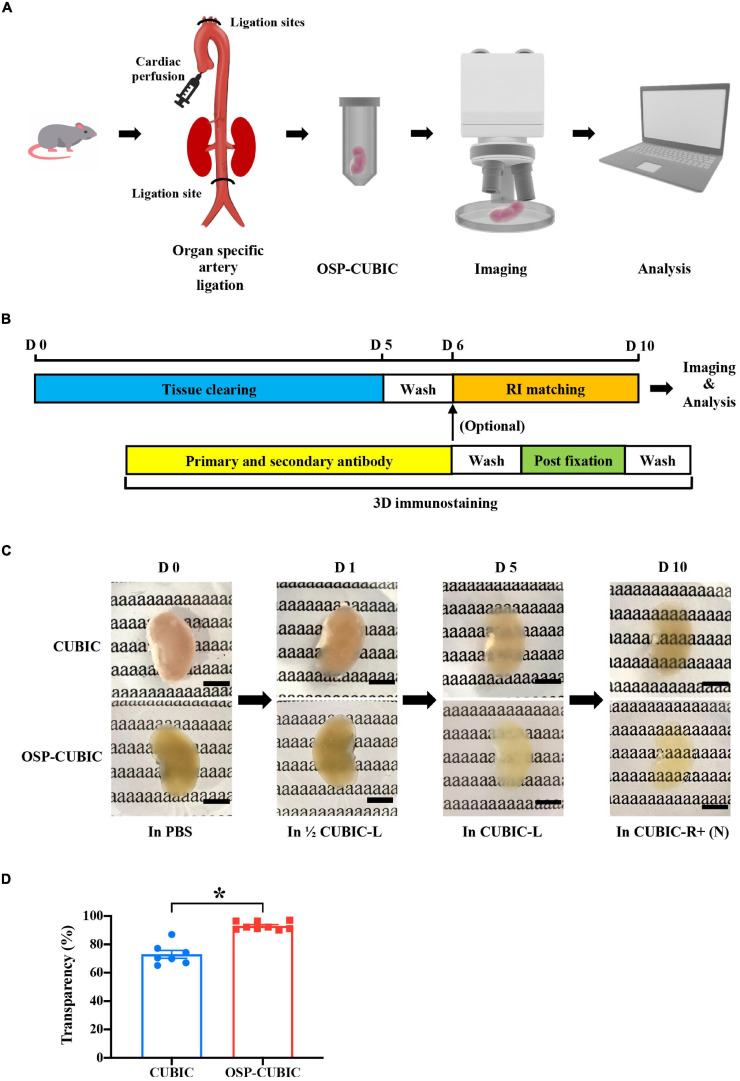
Optimized OSP-CUBIC clearing achieves high transparency of whole-mount kidney. **(A)** Overview of organ-specific perfusion-CUBIC clearing, 3D immunostaining, imaging, and image analysis system. **(B)** Protocol of three major components of OSP-CUBIC: tissue clearing, 3D immunostaining, and RI matching. **(C)** Photos of cleared whole kidney by conventional CUBIC and OSP-CUBIC at different time points. For details of CUBIC-L and CUBIC-R + (N), see Section “Materials and Methods”. Scale bar = 5 mm. **(D)** Quantification of transparency of the kidneys by conventional CUBIC and OSP-CUBIC on day 10 (CUBIC, *n* = 7; OSP-CUBIC, *n* = 9). OSP-CUBIC, organ specific perfusion CUBIC; RI, refractive index; 3D, three-dimensional; PBS, phosphate-buffered saline; D, day. *P < 0.05.

The kidney is a heme-rich organ, and obtaining high transparency is difficult. To develop an efficient tissue clearing protocol for whole-kidney imaging, we began to optimize classic CUBIC tissue clearing methods (Susaki et al., 2015; Matsumoto et al., 2019). We introduced an OSP-CUBIC perfusion protocol to whole-kidney clearing. After ligating the main branch arteries (i.e., inferior renal artery, brachiocephalic trunk, left common carotid artery, and left subclavian artery), we successively perfused various clearing solutions from the left ventricle cardiac apex. The whole kidney was then dissected and immersed in CUBIC-L and CUBIC-R + (N) solutions for complete clearing and subsequent imaging and analysis (Figure 1A). The optimized OSP-CUBIC protocol is composed of three major stages (clearing, 3D immunostaining, and imaging), which takes 10 days (but can vary according to the presence of antibody), for a whole kidney from an adult mouse (Figure 1B). Compared with the conventional CUBIC protocol, the optimized OSP-CUBIC method achieved higher transparency of the adult mouse kidney (Figure 1C). The quantification analysis showed significantly better transparency by OSP-CUBIC (93.06 ± 0.93%, *n* = 9) compared with conventional CUBIC (73.01 ± 2.80%, *n* = 7) on day 10 (Figure 1D; *P* < 0.05). With the aid of the optimized OSP-CUBIC protocol, high transparency was achieved in the adult mouse whole kidney, which is crucial for further kidney imaging and analysis.

### Image Acquisition of Renal Macro- and Microstructures of the Whole Kidney by Light-Sheet Fluorescence and Confocal Microscopy

**FIGURE 2:**
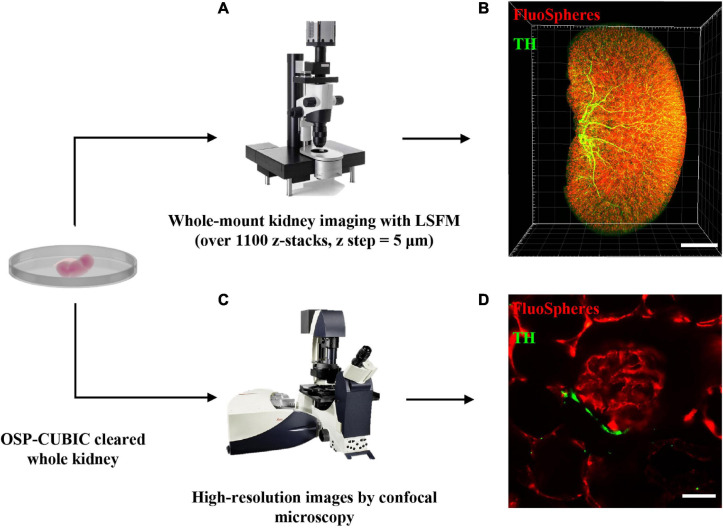
Image acquisition of renal macro- and microstructures of the whole kidney by LSFM and confocal. The whole kidney after OSP-CUBIC clearing, 3D immunostaining, and RI matching was prepared for subsequent imaging. **(A)** Light-sheet fluorescence microscopy was used for whole-mount kidney imaging (over 1100 sections per kidney, z step = 5 μm). **(B)** Three-dimensional reconstruction of the whole kidney, labeled with FluoSphere (microvasculature) and anti-TH antibody (sympathetic nerves). Scale bar = 2 mm. **(C)** To further acquire high-resolution images of local glomerulus and sympathetic innervation, confocal microscopy was used. **(D)** High-resolution imaging of the glomerulus and accompanying sympathetic nerve. Scale bar = 30 μm. LSFM, light-sheet fluorescent microscopy; TH, tyrosine hydroxylase. Sympathetic nerves were labeled with anti-TH antibody (green). The microvasculature was labeled with FluoSphere (red).

After OSP-CUBIC, the optically cleared and 3D-immunostained (sympathetic nerves stained with anti-TH antibody and vasculature labeled with FluoSphere) adult whole kidney was subjected to microscopic imaging (Figure 2). The characteristics of LSFM are beneficial for rapidly acquiring z-stack images and accomplishing high-throughput analyses of the transparent kidney that is labeled with various fluorescence probes. From each kidney sample, over 1100 z-stack images (z = 5 μm) were obtained (Figure 2A). The 3D reconstruction successfully visualized 3D structures of sympathetic nerves, the microvasculature, and the glomerulus in the kidney (Figure 2B and Supplementary Movies 1, 2). For high-resolution details of the renal microvascular structure and accompanying sympathetic innervation, confocal microscopic imaging was performed (Figure 2C), which clearly illustrated sympathetic nerve innervation of the renal microvasculature and glomerulus (Figure 2D and Supplementary Movie 3). 3D imaging after the OSP-CUBIC protocol allowed a comprehensive macroscopic understanding of the kidney microanatomy.

### OSP-CUBIC Clearly Visualizes the Structure of the Renal Microvasculature and Glomerulus

**FIGURE 3:**
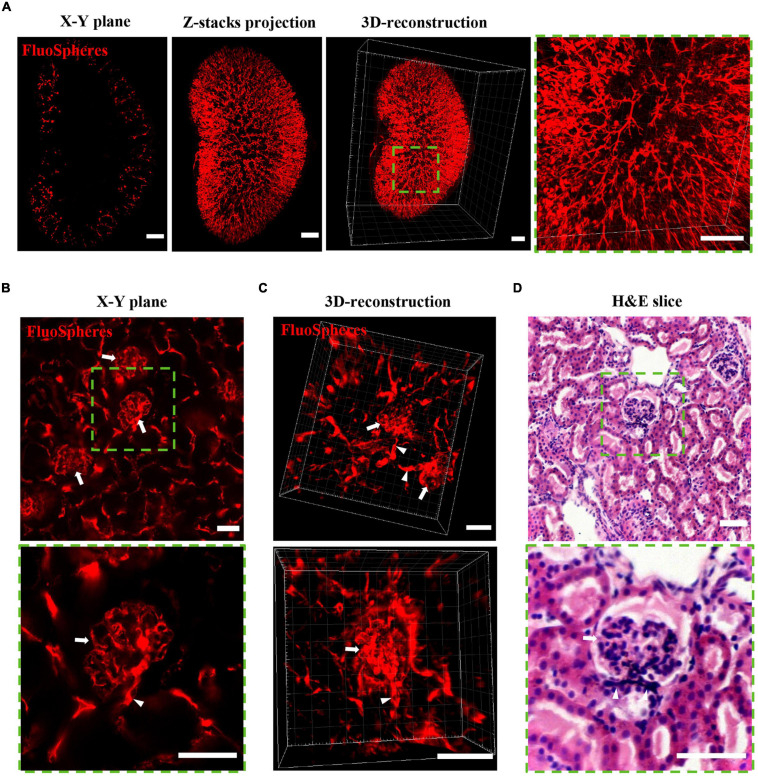
OSP-CUBIC clearly visualizes the structure of the microvasculature and glomerulus. The kidney underwent FluoSphere perfusion or H&E staining. **(A)** *X*–*Y* single-slice plane image, z-stack projection, and 3D-reconstructed of images acquired by LSFM. The 3D reconstruction included 1258 z-section images. Magnification of the square area of the 3D reconstruction showed the dendritic-like renal microvasculature and glomerulus in the distal end. Scale bar = 1 mm. Representative images show the structure of the glomerulus (arrow) and connected afferent/efferent arteriole (triangle) on the *X*–*Y* plane **(B)** and 3D reconstruction **(C)** by confocal microscopy and H&E staining **(D)**. Scale bar = 50 μm. Green square areas are magnified. H&E, hematoxylin-eosin. The microvasculature was labeled with FluoSphere (red).

To visualize the renal microvasculature and glomerulus, the kidney was transcardially perfused with FluoSphere and imaged by LSFM. X-Y plane, z-stack projection, and 3D reconstruction of the kidney showed the distribution of the renal microvasculature and glomerulus across the whole kidney (Figure 3A). Local magnification of the LSFM images clearly showed a dendritic-like microvasculature and distal end glomerulus. Further confocal microscopy illustrated the detailed structure of the glomerulus and connected afferent/efferent arteriole on the *X*–*Y* plane and 3D-reconstructed images (Figures 3B,C and Supplementary Movies 4, 5). H&E staining of the kidney verified the structure of the glomerulus, which was similar to the LSFM and confocal imaging results (Figure 3D). With sufficient clearing, vascular labeling, and 3D imaging, the OSP-CUBIC protocol was clearly able to visualize the renal structure of the microvasculature and glomerulus.

### Three-Dimensional Immunostaining Illustrates the Distribution of Microvascular and Accompanying Sympathetic Nerves

**FIGURE 4:**
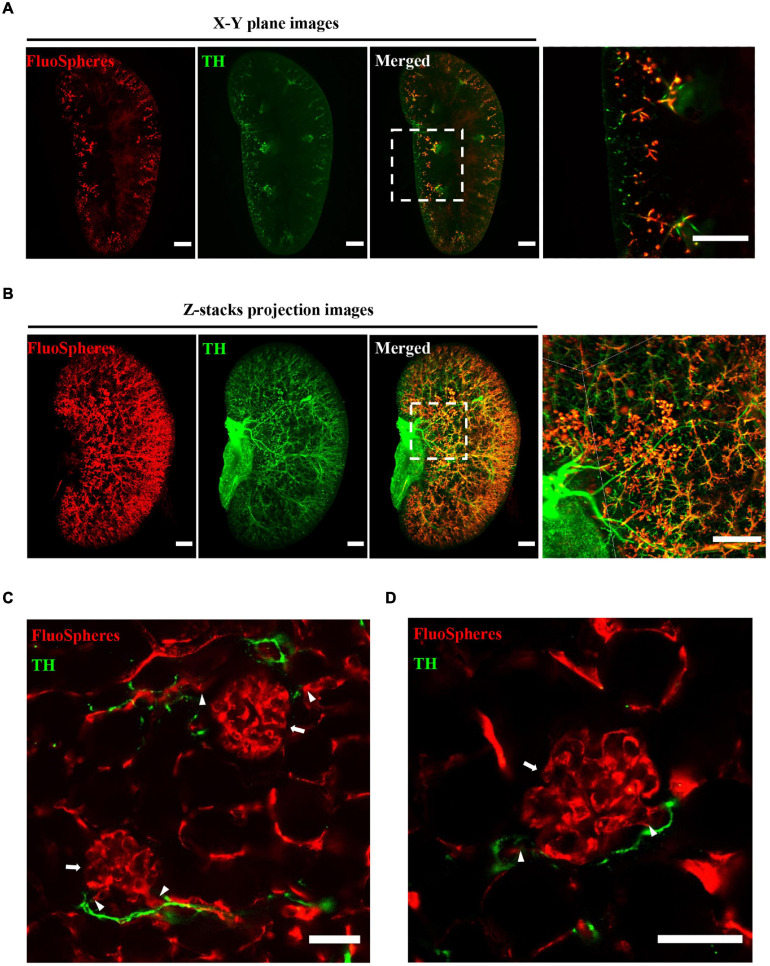
Three-dimensional imaging illustrates the distribution of microvasculature and accompanying sympathetic nerves. The whole kidney was transcardially perfused with FluoSphere and subjected to anti-TH immunolabeling. **(A)** *X*–*Y* plane and **(B)** z-stack projection images showed the distribution of renal microvascular and accompanying sympathetic nerves. The z-stack projection included 1243 z-sections. The square areas are magnified. Sympathetic nerves traveled across the renal microvasculature to the glomerulus. Scale bar = 1 mm. **(C,D)** Representative images of the glomerulus (arrow), connected afferent/efferent arteriole (triangle), and accompanying sympathetic nerves (TH-positive). Scale bar = 50 μm. Sympathetic nerves were labeled with TH (green). The microvasculature was labeled with FluoSphere (red).

The OSP-CUBIC-cleared whole kidney, which had been transcardially perfused with the vascular fluorescent FluoSphere, was subjected to immunofluorescent staining with anti-TH antibody. In whole-mount renal images that were acquired by LSFM, the *X*–*Y* plane (Figure 4A), projection of z-stack images (Figure 4B), and 3D reconstruction (Supplementary Movies 1, 2), TH-stained sympathetic nerves, and the FluoSphere-labeled microvasculature and glomerulus were successfully visualized. Magnified images showed that the sympathetic nerves traveled across the microvasculature to the glomerulus, and most of the microvasculature was innervated by sympathetic nerves. High-resolution 3D imaging by optical sectioning (Figures 4C,D and Supplementary Movie 3) showed that the sympathetic nerves traveled across and wrapped around the renal microvasculature to the position where the afferent/efferent arteriole enters the glomerulus. The sympathetic nerves then merged at the junction of the efferent/afferent arterioles and glomerulus and then continued to travel along the microvasculature to the next glomerulus (Figure 4C and Supplementary Movie 3). Additionally, no sympathetic innervation of the glomerulus was found.

With the aid of OSP-CUBIC clearing, we could see deeper into the kidney. Because of the compatible 3D immunostaining and imaging, we could obtain comprehensive information about the kidney microcirculation and accompanying sympathetic innervation.

### Pressure Overload-Induced Heart Failure and Consequent Renal Dysfunction

**FIGURE 5:**
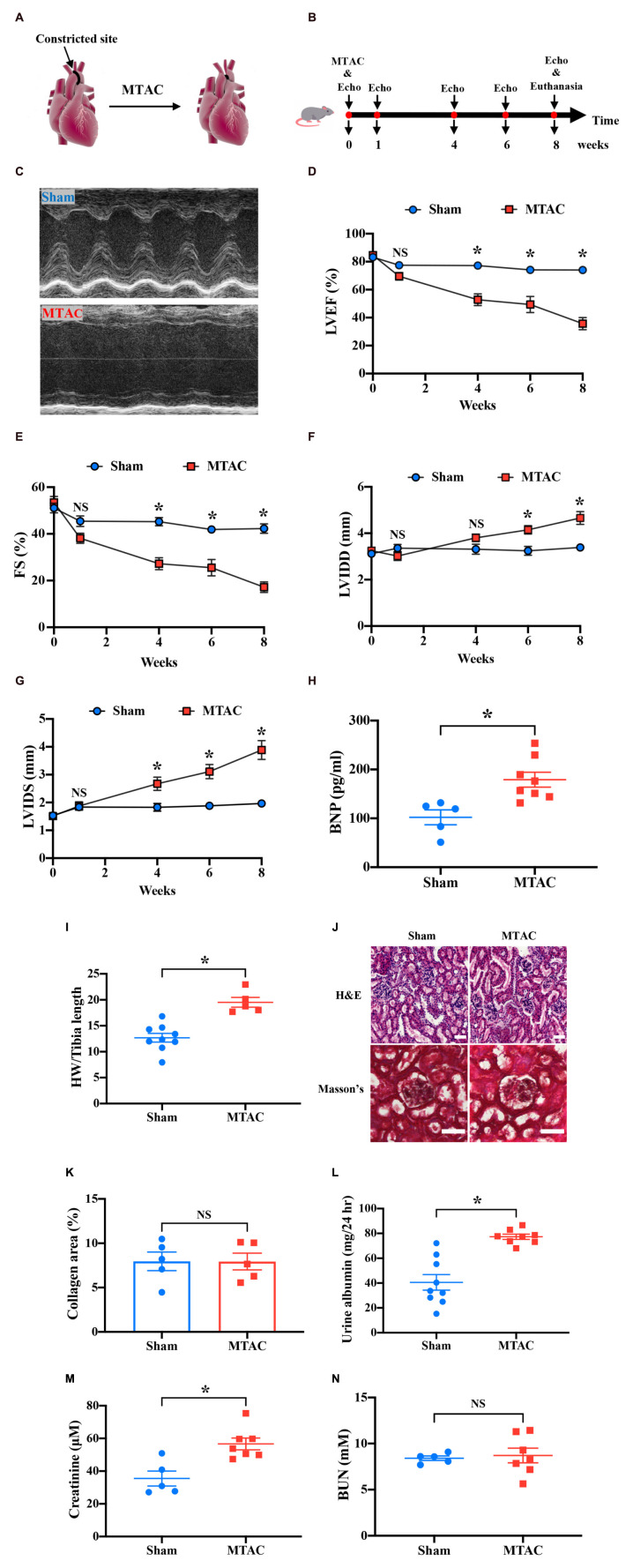
Pressure overload-induced heart failure induces renal dysfunction. **(A)** Overview of minimally invasive transverse aortic constriction (MTAC) protocol. **(B)** Protocol of echocardiography measurement and termination in the MTAC mouse model. The mice underwent the MTAC procedure, and cardiac function was measured by echocardiography at 0, 1, 4, 6, and 8 weeks. After 8 weeks, the mice were euthanized for further study. **(C)** Representative echocardiographic images of mice in the sham group and MTAC group in M-mode. The left ventricular inner diameter significantly increased in the MTAC group. LVEF **(D)** and FS **(E)** decreased in the MTAC group compared with the sham group beginning at 4 weeks (*n* = 8 in sham group, *n* = 6 in MTAC group). LVIDD **(F)** and LVIDS **(G)** in the MTAC group increased at 6 weeks for LVIDD and 4 weeks for LVIDS (*n* = 8 in sham group, *n* = 6 in MTAC group). **(H)** Plasma BNP levels increased in the MTAC group compared with the sham group (*n* = 5 in sham group, *n* = 8 in MTAC group). **(I)** The heart weight-to-tibia length ratio was higher in the MTAC group than in the sham group (*n* = 9 in sham group, *n* = 5 in MTAC group). **(J)** Representative images of H&E staining and Masson’s trichrome between the two groups (*n* = 5 mice per group). Scale bar = 50 μm. **(K)** Percentage of collagen area in Masson’s trichrome kidney section (*n* = 5 mice per group). No significant difference was found between the two groups. **(L)** Twenty-four-hour urine albumin was elevated significantly in MTAC group (*n* = 9 in sham group, *n* = 8 in MTAC group). **(M)** Creatinine levels increased significantly in the MTAC group compared with the sham group (*n* = 5 in sham group, *n* = 7 in MTAC group). **(N)** BUN levels were not significantly different between the MTAC and sham groups (*n* = 5 in sham group, *n* = 8 in MTAC group). *P < 0.05. NS, not significant; MTAC, minimally invasive transverse aortic constriction; Echo, echocardiography; LVEF, left ventricular ejection fraction; FS, fraction shortening; LVIDD, left ventricular inner diameter diastole; LVIDS, left ventricular inner diameter systole; BNP, brain sodium peptide; HW, heart weight; BUN, blood urea nitrogen; H&E, hematoxylin-eosin; hr, hour.

To establish pressure overload-induced HF in mice, an MTAC procedure was performed (Figure 5A). The protocol of the MTAC-induced HF procedure (Figure 5B) was performed, and the LV structure and systolic function were assessed by echocardiography preoperatively and at 1, 4, 6, and 8 weeks post-MTAC.

Representative images of the echocardiography M-mode at 8 weeks showed a greatly prolonged LV inner diameter (LVID) in MTAC mice compared with the sham group (Figure 5C). The M-mode analysis on the parasternal short axis detected a significant decline in systolic function in MTAC mice over 8 weeks (Figures 5D–G). The LV ejection fraction (LVEF) and fractional shortening (FS) were significantly reduced in MTAC mice compared with sham mice (LVEF: 35.68 ± 4.40% *vs*. 74.02 ± 2.33%, *P* < 0.05; FS: 17.19 ± 2.26% *vs*. 42.27 ± 2.02%, *P* < 0.05; MTAC group, *n* = 6; Sham group, *n* = 8) at the endpoint of 8 weeks (Figures 5D,E). After 8 weeks postsurgery, the LVID diastole (LVIDD) and LVID systole (LVIDS) significantly increased in MTAC mice (LVIDD: 4.66 ± 0.28 mm *vs*. 3.39 ± 0.12 mm, *P* < 0.05; LVIDS: 3.88 ± 0.34 mm *vs*. 1.97 ± 0.12 mm, *P* < 0.05; MTAC group, *n* = 6; Sham group, *n* = 8) compared with sham mice (Figures 5F,G). Terminal plasma concentrations of BNP, which increases in humans with clinical HF, were significantly elevated in MTAC mice (179.07 ± 15.22 pg/ml, *n* = 8) compared with the sham group (102.09 ± 15.25 pg/ml, *n* = 5; *P* < 0.05; Figure 5H). The heart weight-to-tibia length ratio also increased in the MTAC group (19.52 ± 0.95 *vs*. 12.68 ± 0.85, *P* < 0.05; MTAC group, *n* = 5; Sham group, *n* = 9; Figure 5I). Altogether, these results confirmed that pressure overload-induced HF was well established in mice.

To assess renal structure in the context of pressure overload-induced HF, we performed renal histological H&E staining and Masson’s trichrome staining of kidney cross-sections (Figure 5J). Quantification of the percentage of fibrotic area showed a similar basal level of interstitial renal collagen and no overt differences between MTAC mice and sham mice (*P* > 0.05; Figure 5K). To further assess renal function, we measured mouse 24-h urine albumin, a commonly used index to assess kidney function, the 24-h urine albumin was increased in MTAC mice (77.30 ± 2.06 mg, *n* = 8) while compared to sham mice (40.57 ± 6.31 mg, *n* = 9; *P* < 0.05; Figure 5L). Furthermore, we detected plasma CRE, a surrogate for a reduction of the glomerular filtration rate, which indicated renal dysfunction at 8 weeks post-surgery. Plasma CRE levels in MTAC mice were elevated compared with the sham group (56.62 ± 3.61 μM *vs*. 35.48 ± 4.58 μM, *P* < 0.05; MTAC group, *n* = 7; Sham group, *n* = 5; Figure 5M). However, plasma BUN was not significantly different between the two groups (*P* > 0.05; Figure 5N). Altogether, these results showed renal dysfunction with increased 24-h urine albumin and plasma CRE level on pressure overload-induced HF before structural and morphological changes of kidney.

### Whole-Kidney 3D Analysis Reveals Renal Sympathetic Hyperinnervation During Heart Failure

**FIGURE 6:**
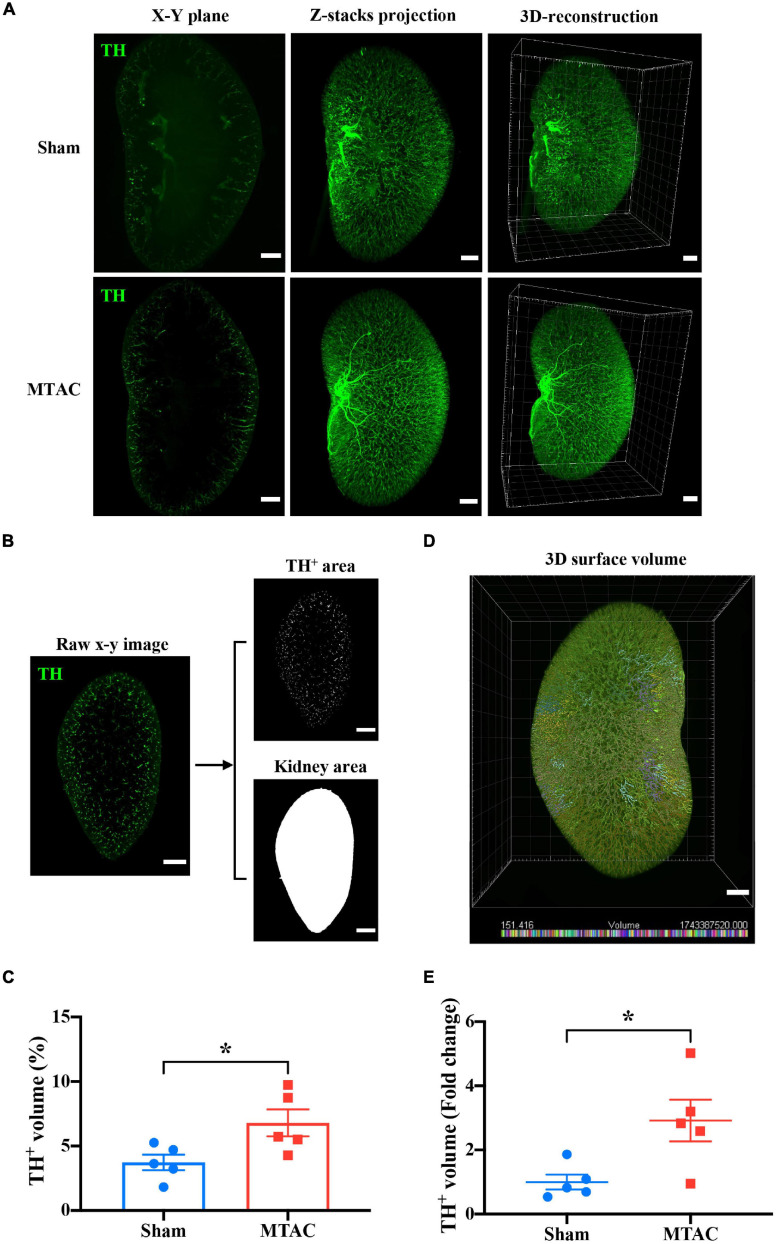
Whole-kidney 3D analysis reveals renal sympathetic hyperinnervation. **(A)** Comparison of sympathetic nerve (TH) innervation in the *X*–*Y* plane, z-stack projection, and 3D construction between the sham group (z-stack of 1258 slices) and MTAC group (z-stack of 1142 stacks). Scale bar = 1 mm. **(B)** The process of calculating the ratio of the TH-positive volume to the total kidney volume is shown. After appropriate threshold binary conversion, the integral area of interest was calculated. Z-step = 5 μm. **(C)** The 3D analysis showed that the sympathetic nerve volume/total kidney volume ratio significantly increased in the MTAC group compared with the sham group (*n* = 5 mice per group). To increase the accuracy of the calculation and for convenience, a 3D surface volume computation protocol **(D)** was introduced. The bottom of the image shows the volume labeled by distinct colors. **(E)** Quantification analysis with real 3D analysis after the 3D surface protocol showed that the TH-positive volume/total kidney volume ratio fold change significantly increased in the MTAC group compared with the sham group (*n* = 5 mice per group). TH, tyrosine hydroxylase. Sympathetic nerves were labeled with anti-TH antibody (green). *P < 0.05.

To further evaluate renal abnormalities under pressure overload-induced HF, we applied OSP-CUBIC-compatible 3D immunofluorescent labeling. Sympathetic nerves of the whole kidney were well labeled with anti-TH antibody. Images of the sympathetic labeled kidney from sham mice and MTAC mice were acquired using LSFM. *X*–*Y* plane images, z-stack projections, and 3D reconstruction clearly showed renal sympathetic distribution in the whole-mount kidney (Figure 6A).

To quantify the sympathetic nerve volume in both groups of mice, an image analysis method was adopted from a previous work (Hasegawa et al., 2019). Briefly, the area of sympathetic nerves and the kidney in the whole-kidney on each the *X*–*Y* plane section were calculated based on threshold-based segmentation. Then, the volumes of objectives (both nerves and kidney) were obtained by the production of total area on each section and sectioning thickness (i.e., z-step of 5 μm) (Figure 6B). The ratio of the sympathetic nerve volume to the whole kidney volume increased in MTAC mice compared with sham mice (MTAC: 6.80 ± 1.04%, *n* = 5; Sham: 3.73 ± 0.60%, *n* = 5; *P* < 0.05; Figure 6C). To avoid potential inaccuracy of nerve fiber segmentation based on 2D section images, we performed 3D segmentation of nerves within the LSFM images using the surface module implemented in a commercial microscopy image analysis software (Figure 6D; see section “Materials and Methods” for details). The quantification of sympathetic nerve volume fold changes showed a significant increase in the MTAC group compared with the sham group (2.92 ± 0.65 *vs*. 1.00 ± 0.23, *P* < 0.05; MTAC group, *n* = 5; Sham group, *n* = 5; Figure 6E). Overall, we found renal sympathetic hyperinnervation at early stages after pressure overload-induced HF, with no structural or morphological alterations.

## Discussion

In the present study, we optimized OSP-CUBIC clearing, whole-mount kidney 3D immunostaining, and high-throughput observation by LSFM and confocal microscopy. We simultaneously visualized the high-resolution 3D structure of the whole-mount renal microvasculature, the glomerulus, and accompanying wrapped traveling sympathetic nerves of the mouse kidney. Further quantification by 3D analysis revealed that renal dysfunction occurred and sympathetic nerve hyperinnervated before structural and morphological changes after pressure overload-induced HF. This study established a novel 3D visualization protocol for kidney research.

It is commonly difficult to acquire in-depth and comprehensive information about the kidney at the whole-mount scale. In a previous study of renal innervation under conditions of kidney disease, to acquire satisfactory images, the researchers performed immunostaining, imaging, and analyses using a half-cut kidney after kidney clearing (Hasegawa et al., 2019). We moved this method forward and achieved high transparency of the whole kidney by OSP-CUBIC. Using this method, the whole-mount kidney was then available for subsequent 3D immunostaining, imaging, and 3D analysis of the whole kidney. A key characteristic of the OSP-CUBIC method was the combination of transcardiac clearing reagent perfusion and organ-specific ligation, which together guaranteed efficient and sufficient whole-kidney clearing both inside and outside the kidney. Additionally, the newly developed OSP-CUBIC protocol is compatible with 3D immunostaining, thereby overcoming the limitation of labeling with genetically engineered animals containing endogenous fluorescent protein. Compared with genetic animals, the use of immunostaining, such as double staining and triple staining, can indicate the distribution and positional relationship of different structures in the same tissue and organ. What is more, genetic animals often have a long production cycle and are expensive, which limits the wide application of tissue clearing technology. In this study, FluoSphere was used for renal microvascular labeling. Compared to other vascular markers, such as isolectin-B4 and CD31, FluoSphere is more suitable for microvascular labeling, and the cost is lower. α-SMA mainly marks the large and medium vasculature surrounded by smooth muscle cells. Further whole-kidney imaging and whole-mount 3D analysis allowed us to achieve a comprehensive macroscopic perspective for future kidney research. The OSP-CUBIC protocol has strong potential for comprehensive 3D analysis of the kidney. However, some issues need to be addressed before this method can be widely applied in kidney research. For example, 3D immunostaining is technically difficult and time-consuming. Various factors, such as the particle radius of antibodies, the concentration of stains, and the incubation time, are involved in 3D immunolabeling (Susaki et al., 2020). Thus, it is difficult to determine the optimal working conditions for each antibody. The development of a more sophisticated 3D staining protocol, such as electro-driven devices, may accelerate the development of whole-mount kidney 3D labeling (Li et al., 2015).

The kidney is abundantly innervated by sympathetic nerves, renal sympathetic nerve fibers release norepinephrine, adenosine triphosphate and neuropeptide Y in the kidney, and the effects of norepinephrine on adrenoceptors most robustly affecting renal function (Bischoff and Michel, 1998; Knight et al., 2003; Osborn et al., 2021). So, sympathetic nerve system contributes to the progression of acute and CKDs (Noh et al., 2020; Grassi et al., 2021). It is found that sympathetic nerves activity is markedly increased in CKD patients, and it is related to renal function independently of comorbidities. Sympathetic activation intensifies as CKD progresses toward kidney failure, and such an intensification is paralleled by a progressive rise in heart rate (Grassi et al., 2021). Various studies using electron microscopy and immunohistochemistry have illustrated the distribution of renal sympathetic nerves fibers (Barajas et al., 1984; Luff et al., 1991, 1992). However, limited by technology, it is challenging to obtain information about whole-mount renal sympathetic nerves. Conventional measurements, such as thin-slice histological examination, often generate biased results. In the present study, the 3D reconstruction of high-resolution images of the microvasculature and glomerulus of the mouse kidney that was subjected to sympathetic nerve immunostaining illustrated that sympathetic nerve traveled across the microvasculature to the glomerulus, and microvasculature was abundantly innervated by sympathetic nerves. These observations are consistent with previous reports that sympathetic nerves play an important role in the functional control of blood vessels in various organs (Yokoyama et al., 2017; Osborn et al., 2021). We also found that sympathetic nerves traveled across and wrapped around the renal microvasculature to a position where the afferent/efferent arteriole enters the glomerulus. Sympathetic nerves then merged at the junction of efferent/afferent arterioles and the glomerulus and then continued to travel along the microvasculature to the next glomerulus, which is consistent with the study that juxtaglomerular renin-secreting granular cells in the region near efferent and afferent arteriole are also innervated by TH positive sympathetic nerves fibers (Luff et al., 1992). In addition, the results also showed that the glomerulus was not innervated by sympathetic nerves. Illustration of the whole-mount renal microvasculature, the glomerulus, and innervated sympathetic nerves allowed us to analyze renal nerve innervation and regulation at the whole-kidney scale.

With regard to CRS, acute and chronic HF is known to cause renal abnormality (Rangaswami et al., 2019). Various theories have been proposed to explain the pathogenesis of CRS, such as prerenal hypoperfusion, RAAS activation, and SNS dysfunction, among others. The SNS is important for homeostatic regulation. Some experimental and clinical studies showed that sympathetic disorder promoted the genesis and maintenance of various diseases, such as HF, hypertension, and CKD (Schiller et al., 2015; Lopes et al., 2020; Veiga et al., 2020). Thus, sympathetic nerves have been considered a potential therapeutic target. For example, renal denervation was performed in patients with resistant hypertension, which showed promising results (Lauder et al., 2020). β-adrenergic blockers are essential for HF therapy, and they have also been evaluated in numerous randomized controlled trials. β blockers have been shown to improve New York Heart Association (NYHA) class and LVEF, alleviate symptoms, reduce hospitalization burden, and prolong survival (Ghali et al., 2009; Castagno et al., 2010; Badve et al., 2011; Rangaswami et al., 2019). However, it is difficult to determine the mechanisms by which sympathetic nerve therapy exerts its beneficial effects. Conventional methods that are used to assess sympathetic nerve function rely on plasma or tissue catecholamine measurements, which makes it difficult to accurately assess the sympathetic nerve innervation of specific organs. Thus, there is currently no unified understanding of the role of sympathetic nerves in the pathogenesis of kidney diseases, especially in the setting of HF. In the present study, we quantified whole-kidney sympathetic nerve innervation volumes based on whole-kidney 3D analysis and found that pressure overload-induced HF led to renal sympathetic hyperinnervation. In a previous study on kidney ischemia-reperfusion model, the results showed decreased sympathetic nerves innervation (Hasegawa et al., 2019). As kidney ischemia–reperfusion contributes to acute kidney injury, the model we established in this study is chronic kidney injury model. In the setting of HF, the kidney is hypo-perfused, which may act to upregulate the sympathetic nerves by activating the RAAS. Limited by technology, tissue or plasma norepinephrine levels were not measured; we detected TH innervation in the kidney instead, as TH is not only a molecular marker of sympathetic nerves but also a key enzyme for norepinephrine synthesis. The increase of renal TH expression can indirectly indicate the increase of norepinephrine production and sympathetic activation. Our findings support the emerging notion that dysregulation of the SNS plays an important role in early stages of cardiac-renal interactions (Rangaswami et al., 2019). More studies are needed to illustrate the involved molecular mechanism in sympathetic mediated renal function regulation.

## Conclusion

We optimized a whole-mount kidney OSP-CUBIC clearing, 3D-immunostaining, image acquisition, and 3D analysis system and successfully visualized the 3D structure of the renal microvasculature, the glomerulus, and accompanying sympathetic nerves. We observed sympathetic hyperinnervation at early stages after pressure overload-induced HF in mice. This newly developed whole-organ tissue clearing and imaging method has great potential for further studies of kidney diseases and can provide comprehensive information at the whole-mount scale. This method can also be performed with other heme-rich organs, such as the heart, for which achieving satisfactory clearing and imaging of the whole organ is difficult.

## Data Availability Statement

The original contributions presented in the study are included in the article/Supplementary Material. Further inquiries can be directed to the corresponding author/s.

## Ethics Statement

The animal study was reviewed and approved by China-Japan Friendship Hospital.

## Author Contributions

CW performed the primary experiments, acquired and analyzed the data, and wrote the manuscript. FY and ML performed some of the LSFM imaging and image processing. YT, ZG, YC, YW, and QL performed some of the animal experiments. CY, YF, and MW designed some of the experiments. JZ, XL, YG, and WK equally designed the study, analyzed the data, and wrote the manuscript. All authors contributed to the article and approved the submitted version.

## Conflict of Interest

The authors declare that the research was conducted in the absence of any commercial or financial relationships that could be construed as a potential conflict of interest.
