# Supplementary material for: Whole-Mount Kidney Clearing and Visualization Reveal Renal Sympathetic Hyperinnervation in Heart Failure Mice
Source: Front Physiol. 2021 Jul 8;12:696286. doi: 10.3389/fphys.2021.696286 (PMC8297621; doi:10.3389/fphys.2021.696286)
Supplement: Supplementary file 6 [file Data_Sheet_1.docx]

Supplementary Material

**Supplementary Movies 1.** **Three-dimension (3D) reconstruction of LSFM-imaged whole-kidney microvasculature and sympathetic nerves.** The whole kidney was transcardially perfused with FluoSphere and then subjected to anti-tyrosine hydroxylase (TH) antibody labeling. The z-stack 3D reconstruction showed the distribution of whole renal microvasculature and accompanying sympathetic nerves. Sympathetic nerves were labeled with anti-TH antibody (green). The microvasculature was labeled with FluoSphere (red). LSFM, light-sheet fluorescent microscopy.

**Supplementary Movies 2.** **Slice-by-slice LSFM scan shows renal vasculature and accompanying sympathetic nerves.** The kidney was subjected to FluoSphere vascular staining and anti-tyrosine hydroxylase (TH) antibody immunolabeling. Whole-mount slice images showed the distribution of renal microvasculature, distal glomerulus, and accompanying sympathetic nerves. Sympathetic nerves were labeled with anti-TH antibody (green). The microvasculature was labeled with FluoSphere (red).

**Supplementary Movies 3.** **High-resolution kidney microvasculature, glomerulus, and innervated sympathetic nerves imaged by confocal microscopy.** For high-resolution details of the renal microvascular structure and accompanying sympathetic innervation, confocal microscopic imaging was performed. The high-resolution 3D imaging by optical sectioning illustrated microvascular, glomerulus, and innervated sympathetic nerves. Sympathetic nerves were labeled with anti-TH antibody (green). The microvasculature was labeled with FluoSphere (red).

**Supplementary Movies 4.** **Slice scan illustrates the network of renal microvasculature and distal glomerulus.** The kidney underwent FluoSphere perfusion and was imaged by confocal microscopy. The slice-by-slice scan clearly illustrated the dendritic-like renal microvasculature and distal end glomerulus. The microvasculature was labeled with FluoSphere (red).

**Supplementary Movies 5.** **Three-dimensional reconstruction shows the structure of the glomerulus and connected afferent/efferent arteriole.** The kidney was transcardially perfused with FluoSphere and visualized by confocal microscopy. The 3D reconstruction of the glomerulus shows the high-resolution glomerulus and connected afferent and efferent arteriole. The microvasculature was labeled with FluoSphere (red).
